# Career Education Skills and Career Adaptability among College Students in China: The Mediating Role of Career Decision-Making Self-Efficacy

**DOI:** 10.3390/bs13090780

**Published:** 2023-09-19

**Authors:** Xinqiao Liu, Xinyuan Zhang, Yiming Dang, Wenjuan Gao

**Affiliations:** 1 School of Education, Tianjin University, Tianjin 300350, China; 2 Institute of Higher Education, Beihang University, Beijing 100191, China; 3 School of Public Administration, Beihang University, Beijing 100191, China; 4 Research Center for Reform and Development of Graduate Education, Beijing 100191, China

**Keywords:** career education skills, career adaptability, college students, mediation model

## Abstract

In the past, the shift in career patterns and the unprecedented disruptions caused by events such as COVID-19 have posed notable challenges for job seekers. This holds particularly true for college students who are preparing to enter the workforce. In this context, enhancing career adaptability plays a vital role in shaping their career development. The primary objective of this research was to investigate the relationship between career education skills and career adaptability among 273 undergraduate students in China. Additionally, the study aimed to explore the mediating effect of career decision-making self-efficacy in shaping this relationship. The findings of the correlation analysis indicate a significant positive correlation between career education skills and career adaptability. Moreover, the results of the mediation model revealed that career education skills significantly contribute to improving career adaptability along with the mediating effect of college students’ self-efficacy in making career decisions. This study suggests that universities should prioritize the development and expansion of career education initiatives. They should not only help establish clear career goals for college students but also cultivate a positive and flexible career outlook to assist them in better adapting to various changes that may arise throughout their career journeys.

## 1. Introduction

The traits of varied, limitless, and flexible career paths are gaining more prominence in the current worldwide community [1]. The pressure brought by the uncertainty and unpredictability of career prospects requires individuals to continuously make emotional and cognitive adjustments [2,3,4]. The global spread of COVID-19 has resulted in numerous career interruptions that have contributed to a more challenging job market for upcoming professionals and heightened anxiety among university students [5,6,7,8,9,10]. In a job search process full of uncertainty, college students are not only required to acquire relevant job skills but also need to continuously evaluate and adapt to their career paths [11,12,13]. Research has been carried out to demonstrate that seeking employment is a self-regulatory procedure [14]. Among Chinese college graduates, career adaptability is a crucial element in achieving a successful job search, which enhances their job prospects and compatibility [15], thus advancing their career growth.

Career adaptability was initially introduced in the 1950s as a concept that pertains to the tools and strategies that people employ to proficiently handle the difficulties and accountabilities linked with their present and future job positions [16]. The capacity to adjust to the work environment results from a blend of attitudes, skills, and actions [17,18], which can assist people in effectively handling the challenges and modifications they face in their professions [19]. Career adaptability comprises four self-regulatory traits: concern, control, curiosity, and confidence. Individuals who possess strong career adaptability can anticipate future events (concern) [20], make independent choices and take accountability for their own growth (control) [21], investigate and shape their identity in diverse circumstances and positions (curiosity) [22], and have faith in their capacity to fulfill personal goals and surmount occupational obstacles (confidence) [17]. Simultaneously, a significant amount of research has shown the significance of adaptability in careers within the field of career advancement. Career adaptability, which is a fundamental idea in career construction theory [18,23,24], has a strong and positive correlation with both job search achievement [25,26] and job contentment [27,28,29,30]. Improving the adaptability of students’ careers can assist not only in a successful transition from school to work [31,32] but also in better preparation for careers [33], informed career decisions [34], and a greater chance of career success [29,35]. Furthermore, high levels of career adaptability can reduce individuals’ turnover intentions [28,36] and enhance their promotability [37,38]. Hence, it is imperative to investigate and elucidate how career flexibility can be improved among university attendees.

College serves as a crucial transitional phase for students, bridging the gap between career exploration and career establishment [39,40,41]. It represents a pivotal period for the transition from college to career and job preparation [31]. Nonetheless, this change can frequently trigger emotions of doubt and vulnerability, which may impact students’ mental health [42]. Adequate career preparedness equips students with the knowledge to identify and seize various opportunities while acquiring skills that contribute to career success [43]. The arrangements have a notable impact on initial professional encounters, which consequently affect future employment changes and the durability of one’s career [44,45]. Unfortunately, career education research in China started relatively late compared to developed Western countries, and the coverage of career education courses remains incomplete. It should be emphasized that the lack of thorough career education leads to students not having a clear understanding of themselves, a limited comprehension of the job market, and insufficient readiness for job hunting. This comprises inadequate self-appraisal, occupational information, goal selection, planning, and problem-solving skills. Career adaptability in college students is closely linked to their career decision-making self-efficacy, which is made up of crucial elements. Career exploration and decision-making behavior are significantly influenced by self-efficacy in career decision-making [46], which is also a critical factor in maintaining a successful career trajectory [47]. The main objective of this research was to examine the direct and indirect relationships between the three factors. On this basis, we especially explored the mediating role of career decision-making self-efficacy between career education skills and career adaptability among Chinese university students. The results not only improved our comprehension of the relationship among these factors but also offer practical observations for academic policymakers, college executives, and instructors to apply more specific strategies to foster the future growth of university students.

### 1.1. Career Education and Career Adaptability

Savickas’ career construction theory suggests that career adaptability is influenced by a combination of internal and external factors, including personality traits and environmental circumstances [18]. This perspective has been corroborated by numerous studies. Regarding internal factors, a proactive personality [48,49,50], the Big Five personality traits [51], future orientation [52], core self-evaluation [53], and others have predictive effects on career adaptability. For the external aspect, support from parents and teachers [54,55], role modeling [56], and access to career education [57] also play significant roles in shaping the development of career adaptability. As mentioned previously, the significance of career development cannot be understated, and yet there remains a dearth of supporting evidence in this domain.

As a paradigm of career intervention, career education is an endeavor that utilizes predictable developmental paths to foster personal attitudes and skills [58]. In the context of higher education, career education is defined as education aimed at career development, which means helping students manage the ever-changing sequence of roles in their family, school, community, profession, and leisure [59]. This is primarily facilitated through career education courses, career counseling services, and other means to promote their career development [59,60,61]. This effect has been confirmed by several studies. For example, in a study conducted with biomedical doctoral students, researchers designed and implemented a course named “Effective Career Planning for PhDs”. According to the findings, the inclusion of career planning in the doctoral program aided students in gaining knowledge about the job market and personal situations, resulting in a more optimistic outlook toward their career paths [62]. This suggests an enhancement in career apprehension. Dozier et al. assessed the pessimistic career musings of university attendees before and after the introduction of a vocational program, discovering that it ameliorated their negative career contemplations [63], fostering inquisitiveness toward their careers. Gu et al. offered career intervention programs to 413 Chinese high school students, and the findings of a questionnaire survey they conducted revealed that these programs significantly improved students’ ability to make career decisions [64], indicating that students exhibited more career confidence. In summary, career education is a whole aggregation consisting of individual perception learning and career planning learning. After conducting a review of the literature, it became apparent that there is a scarcity of empirical research on career education for college students in China. Specifically, there has been little investigation into the direct impact of career education skills on career adaptability in China. Consequently, there is a necessity to extend and intensify this investigation.

### 1.2. Mediating Role of Career Decision-Making Self-Efficacy

Self-efficacy is mainly built through four key factors, as stated in social cognitive theory: mastery experiences, vicarious experiences, verbal persuasion, and emotional and physical states [65]. Social cognitive theory is applied in the field of careers to elucidate how people arrive at career or vocational choices. Career decision-making self-efficacy refers to the confidence or belief an individual has in their capability to make career decisions as defined by sources [65,66,67]. The five dimensions of the system are self-appraisal, occupational information, goal selection, planning, and problem solving [68]. Several studies have shown a strong correlation between self-efficacy in making career decisions and engaging in career-oriented tasks [69]. Among other factors, greater career exploration [47], reduced decision-making hesitation [60], fewer difficulties in making career decisions [70,71], and a perceived increase in career optimism [72] are linked to higher levels of self-efficacy in career decision making. Numerous studies have revealed a favorable association between self-efficacy in making career decisions and the ability to adapt to different careers. Most of the research suggests that there is a favorable correlation between self-efficacy in making career decisions and the ability to adapt to different career situations [73,74]. This implies that career decision-making self-efficacy can be viewed as a flexible reaction that affects an individual’s career adaptability [75]. Some studies have identified career decision-making self-efficacy as a mediating factor that simultaneously leads to career adaptability. A study conducted on 205 undergraduates of a university in Malaysia revealed that self-efficacy in career decision making acted as a mediator between emotional intelligence, self-respect, and career adaptability, indicating a robust linear correlation with career adaptability [76]. A different study involving 472 university students from the UK revealed that the ability to make career decisions was significantly influenced by emotional intelligence through career decision-making self-efficacy [70]. Career adaptability was influenced by social support through the mediation of career decision-making self-efficacy in a study that followed 145 undergraduate students from China over time [77,78]. Furthermore, there exists a limited amount of written works that have explored the favorable influence of career education on career decision-making self-efficacy. For instance, a managed trial demonstrated that the career decision-making self-efficacy of college students was enhanced in general after they finished a course on career development [79,80]. Nonetheless, there is a dearth of studies on the direct mechanisms that connect career education skills and career adaptability, especially among Chinese undergraduates.

Based on the literature and our observations, we speculate that implementing career education can enhance students’ career decision-making self-efficacy, thereby improving career adaptability. In other words, career decision-making self-efficacy acts as a mediating factor through which career education skills influence career adaptability.

### 1.3. Research Hypotheses

In order to promote social harmony and stability, it is essential for college students to possess career education skills and career adaptability, as these factors play a critical role in their successful employment. Exploring the mediating role of career decision-making self-efficacy among college students is necessary to understand the relationship between career education skills and career adaptability, as it actively influences career activities. Nonetheless, the existing research does not give enough attention to the influence of career education skills on career adaptability and the intervening function of career decision-making self-efficacy, particularly in the Chinese educational setting. To bridge this research gap, promote educational reforms, and enhance the competitiveness of college students in the labor market, we proposed the following research hypotheses based on the literature, theoretical foundations, and research objectives.

**Hypothesis** **1.** *There is a significant positive relationship between college students’ career education skills and career adaptability.*

**Hypothesis** **2.** *There is a significant positive relationship between college students’ individual perception and career planning and their concern, control, curiosity, and confidence.*

**Hypothesis** **3.** *Career decision-making self-efficacy plays a mediating role in the relationship between college students’ career education skills and career adaptability.*

**Figure 1:**
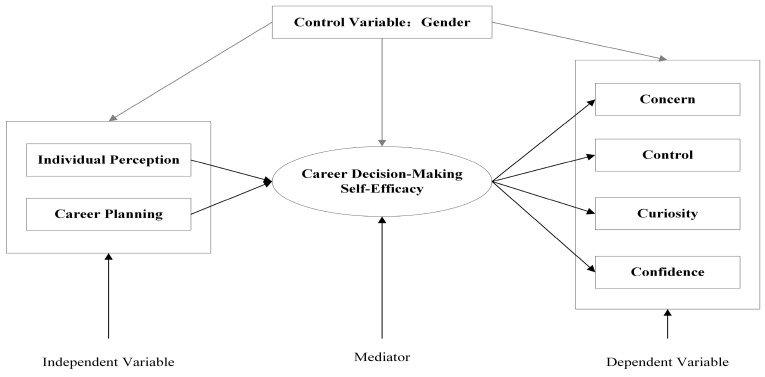
Theoretical research framework.

Based on relevant literature and theories, we established a theoretical research framework as shown in Figure 1.

## 2. Materials and Methods

### 2.1. Participants

The data used in this study were collected through a random sampling survey method at the T University. The survey was open to all undergraduate students for a period of one month. The survey questionnaire gathered information on demographic factors, the level of career self-development, the level of career adaptability development, and level of career decision-making self-efficacy development. After excluding questionnaires with irregular patterns or incomplete responses, a total of 222 valid questionnaires out of the 273 collected were subjected to empirical analysis. Female participants accounted for 55.9%, while male participants accounted for 44.1%. The sample included 13.5% first-year college students, 42.3% second-year students, 16.7% third-year students, 25.7% fourth-year students, and 2.3% five-year students.

### 2.2. Measures

Career education skills: In this study, the assessment of the career education skills was conducted through two dimensions: individual perception and career planning. Cronbach’s alpha yielded a reliability coefficient of 0.9063 for the entire scale.

Career adaptability: The Career Adapt-Abilities Scale–Short Form (CAAS-SF) was utilized to evaluate the degree of adaptability in careers among university students. The scale is composed of four categories: concern, control, curiosity, and confidence [81]. Each dimension included three indicators. The scale demonstrated a Cronbach’s alpha of 0.9317, indicating high reliability.

Career decision-making self-efficacy: The Career Decision-Making Self-Efficacy Scale–Short Form (CDMSE-SF) was utilized to gauge the degree of career decision-making self-efficacy in college students during this research. The measurement tool comprises five categories: self-appraisal, occupational information, goal selection, and planning, and problem solving [82]. The scale exhibited a Cronbach’s alpha of 0.9373, indicating high overall reliability.

All three of these scales were positively scored using a five-point Likert scale, with higher scores indicating better career education skills, higher career adaptability, and higher career decision-making self-efficacy among college students, respectively.

### 2.3. Data Analysis

First, we utilized Stata17 to analyze and present the descriptive statistics of the career education skills, career adaptability [55], and career decision-making self-efficacy among the college students. Next, we conducted a bivariate correlation analysis to examine the relationships among career education skills, career adaptability, and career decision-making self-efficacy. Finally, using Mplus7.4 and maximum likelihood estimation, we established a mediation model to examine the mediating effect of career decision-making self-efficacy on the relationship between career education skills and career adaptability. There were no missing values in the data collected for the study.

## 3. Results

### 3.1. Descriptive Statistics

The descriptive statistics for the dimensions of career education skills, career adaptability, and career decision-making self-efficacy are presented in Table 1. The table includes information on the mean, standard deviation, minimum, maximum, skewness, and kurtosis. The analysis revealed that the mean score for career education skills was 3.258, which was higher than the median, suggesting that the college students had accomplished a positive learning result in this field. Specifically, the individual perception dimension had an average value of 3.539, while the career planning dimension had an average value of 3.057. Specifically, the execution of career education exhibited comparatively inferior outcomes in the field of career planning. Career adaptability had an average value of 3.752, exceeding the median. The college students exhibited a well-rounded career adaptability with similar scores in the four dimensions of career concern, control, curiosity, and confidence, indicating a high level of development [83]. Career decision-making self-efficacy had an average value of 3.535, which was higher than the median. The college students demonstrated self-assurance in their capacity for self-appraisal, occupational information, goal selection, planning, and problem solving.

### 3.2. Correlation Analysis

Table 2 displays the correlation matrix depicting the relationships between career education skills (individual perception and career planning), career adaptability (career concern, career control, career curiosity, and career confidence), and career decision-making self-efficacy among college students. The correlation coefficients among the variables were significant at the *p* < 0.01 level. First, a significant positive correlation was observed between career adaptability and career education skills (r = 0.596, *p* < 0.01), highlighting the importance of career education skills in fostering students’ adaptive capacities in their career development. Second, a significant positive correlation was found between career decision-making self-efficacy and career education skills (r = 0.633, *p* < 0.01), suggesting that higher levels of career education are associated with greater confidence in making career decisions. Similarly, a significant positive correlation was identified between career decision-making self-efficacy and career adaptability (r = 0.785, *p* < 0.01), indicating that individuals with stronger self-efficacy in career decision-making were more likely to exhibit higher levels of adaptability in their careers. Moreover, gender showed a significant positive correlation with career decision-making self-efficacy (r = −0.129, *p* < 0.1), particularly in the fields of self-appraisal, occupational information, and planning. This suggests that gender differences may influence certain aspects of career decision-making self-efficacy for college students.

### 3.3. Mediation Model

**Figure 2:**
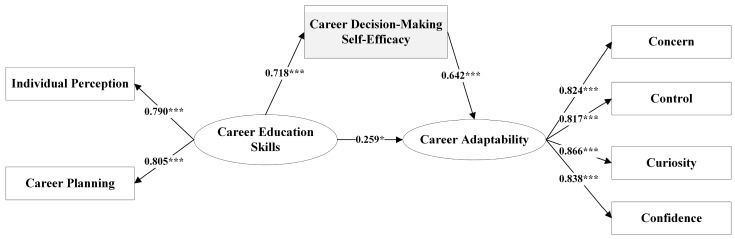
Mediation effect model. Note: *** *p* < 0.01, * *p* < 0.1.

After accounting for gender as a control variable, we employed a mediation model and utilized the bootstrap method to examine the mediating role of career decision-making self-efficacy between career education skills and career adaptability. It is generally recognized that a cutoff value close to 0.95 for TLI and CFI and a cutoff value close to 0.08 for SRMR are needed before concluding that there is a relatively good fit between the hypothesized model and the observed data [84]. Consequently, the model fit indices indicated a satisfactory goodness of fit (CFI = 0.972, TLI = 0.956, SRMR = 0.045). The results of the model are presented in Table 3. In the mediation model, the direct effect of career education skills on career adaptability was estimated to be 0.259 (R^2^ = 0.518, *p* = 0.052) with a confidence interval of [0.025, 0.559], indicating a significant effect of career education skills on career adaptability at the 10% level. The indirect effect of career decision-making self-efficacy between career education skills and career adaptability was 0.461 (R^2^ = 0.721, *p* < 0.01) with a confidence interval of [0.286, 0.647]. These results present a significant indirect effect, indicating that career education skills have a direct positive impact on career adaptability, and this relationship is further strengthened by the indirect effect mediated through career decision-making self-efficacy. Figure 2 visually illustrates the results of the mediation analysis.

## 4. Discussion

The objective of this study was to examine how career education skills affect career adaptability in Chinese college students while also analyzing the mediating impact of career decision-making self-efficacy. In the context of Chinese education, the relevant theories and the relationship between these variables are better understood due to the research findings. Additionally, these resources offer significant proof for decision makers in education, administrators at universities, and educators to enact more precise strategies in fostering the professional growth of undergraduate students.

Career education skills and the average value for individual perception and career planning dimensions are moderately high, as indicated by the results of descriptive analysis. These findings indicate that Chinese college students generally have a favorable exposure to career education. The results were consistent with a survey carried out by Scripps Research, which emphasized the beneficial effects of career education on improving people’s self-awareness and ability to plan their careers [62]. However, compared to individual perception, the mean of career planning was slightly lower, suggesting that Chinese college students may have relatively limited awareness and skills in career planning. This disparity could be attributed to the prevalent exam-oriented education culture in China, where emphasis is often placed on academic performance, overshadowing the exploration and strategic planning of future career paths [85,86].

The correlation analysis results validated a noteworthy affirmative correlation between career education skills and career adaptability, supporting the hypothesis that human capital enhances career adaptability [17,73]. Career adaptability had a strong positive correlation with both the dimensions of individual perception and career planning, which together make up career education skills. The discovery affirmed the idea that adaptability in one’s career is impacted by both internal factors (personal perception) and external factors (career planning) [18]. Career adaptability, as per career construction theory [87], is marked by various dimensions such as planning, exploration, and decision-making, which are also signs of career maturity [23]. This implies that we can foster students’ capacity for self-discovery and vocational strategizing by employing diverse methods to aid them in making career choices and improving their career adaptability. Moreover, the research uncovered noteworthy affirmative associations amid career education skills and the aspects of career adaptability, particularly among all dimensions, which additionally bolsters the career construction hypothesis.

This research presented additional proof that career decision-making self-efficacy acts as a mediator between career education skills and career adaptability in Chinese college students, offering fresh perspectives in the literature. The study showed that career education skills had a significant positive effect on career adaptability development at a 10% significance level when controlling for gender using the mediation model. The findings of this study are consistent with previous research [57]. Moreover, the research indicated that career education skills impact the growth of career adaptability by enhancing one’s career decision-making self-efficacy. Previous studies [70,76,77] have reported that career education courses have a positive impact on career decision-making self-efficacy [79], which in turn plays a mediating role in career adaptability. According to social cognitive theory and career construction theory [23,65,83], career education can increase self-efficacy in making career decisions by offering experiential learning and verbal communication that provide students with encouragement, evaluation, advice, and persuasion. In the meantime, enhanced self-efficacy in making career choices stimulates people’s drive, mental abilities, and adherence to a range of tasks necessary for successful career decision-making. Consequently, this leads students to actively participate in career planning [88], actively explore their career environment and opportunities, and ultimately improve their career adaptability by making better career choices.

Moreover, our discoveries carry substantial theoretical and pragmatic consequences. This study enhances the comprehension of the favorable impact of career education skills on career adaptability from a theoretical standpoint. It fills a void in prior research that disregarded the significance of career education skills as a determinant of career adaptability. Additionally, examining the fundamental processes by which career education skills influence career adaptability offered fresh factual proof in favor of career construction theory. On a practical level, this study provides insights for educational authorities in terms of specific educational practices. First, educational authorities should strengthen guidance and supervision of the effectiveness of career education in schools. It is crucial to establish dedicated teams to plan and oversee career education initiatives, making it an integral component of talent development goals. To aid students in tackling career planning and decision-making challenges, universities must furnish a variety of career education resources, including classes, organizations, and talks, and actively encourage their use [89]. Second, by fostering flexible career perspectives, universities can assist students in navigating complex and evolving career landscapes more effectively.

## 5. Limitations

First, the data were acquired through self-report surveys, which have the potential to introduce response biases.

Second, caution should be exercised when generalizing the research findings to a larger population, as the survey participants in this study were limited to only one university in China. To improve the generalizability of the study, upcoming investigations should strive to incorporate individuals from a variety of educational establishments, geographic locations, levels of education, and fields of study.

Third, the study currently in use employed cross-sectional data, which established the correlation, but it did not allow for causal inferences or exploration of the temporal dynamics between these variables. To overcome this constraint, it would be beneficial to employ a combination of qualitative and quantitative tools for in-depth analysis, drawing inspiration from research conducted by Parmentier et al. [90] and Romero-Rodriguez et al. [91].

Finally, the findings pertain to a cohort of students who exhibited particular career education patterns, influencing their individual perceptions and career planning. However, our current understanding remains incomplete, as we lack insight into the patterns and connections that may arise among students who have not participated in career education. To rectify this limitation, subsequent studies will be conducted to gather data from students who have not been exposed to career education, thus establishing a control group for comparative analysis.

## 6. Implications of Educational Practice and Conclusions

### 6.1. Implications of Educational Practice

This study has important implications and applications for educational practice. 

First, career education skills among Chinese university students have a positive effect on career adaptability. Therefore, we recommend that educational authorities and teachers prioritize career education skills for college students [92]. It is crucial to align talent development goals with the evolving demands of the job market and society.

Second, career decision-making self-efficacy can act as a facilitating mediator in promoting career adaptability. Institutions can enhance career decision-making self-efficacy by improving career education skills and strengthening students’ abilities and beliefs so that students can sustain the development of their knowledge, skills, and mindset for successful career decisions and be fully prepared for their future career path.

Third, we should give full attention to students’ career self-development by utilizing various means and methods, such as the career education mentioned in this paper, to enhance students’ career adaptability. By increasing their career decision-making self-efficacy, more career exploration and preparation will be carried out by individuals [47,72], and comprehensive and all-around improvements can be achieved.

### 6.2. Conclusions

First, the study revealed that Chinese college students who receive career education have a higher level of career adaptability, as there was a notable positive relationship between career education learning and career adaptability development. Moreover, career concern, career control, career curiosity, and career confidence were significantly positively correlated with the subdimensions of career education skills, individual perception, and career planning.

Second, the research indicated that Chinese university students who acquire career education experience an advantageous effect on their ability to adjust to their career along with the crucial involvement of self-efficacy in making career decisions. This highlights the importance of empowering students with the necessary skills and confidence to make informed career decisions.

## Figures and Tables

**Table 1:** Descriptive statistics analysis results.

		N	Mean	Standard Deviation	Min	Max	Skewness	Kurtosis
Career education skills	Individual perception	222	3.539	0.679	1	5	−0.549	3.792
	Career planning	222	3.057	0.808	1	5	−0.239	2.708
	Total	222	3.258	0.687	1	5	−0.213	3.191
Career adaptability	Concern	222	3.718	0.762	1	5	−0.25	2.988
	Control	222	3.760	0.684	1	5	−0.304	3.68
	Curiosity	222	3.763	0.697	1	5	−0.395	3.644
	Confidence	222	3.769	0.706	1	5	−0.308	3.421
	Total	222	3.752	0.626	1	5	−0.35	4.226
Career decision-making self-efficacy	Self-appraisal	222	3.536	0.654	1	5	−0.225	3.775
	Occupational information	222	3.483	0.645	1.4	5	0.079	3.182
	Goal selection	222	3.528	0.615	1.2	5	−0.181	4.088
	Planning	222	3.549	0.649	1.2	5	−0.166	3.641
	Problem solving	222	3.591	0.613	1.5	5	−0.119	3.485
	Total	222	3.535	0.559	1.5	5	−0.062	3.884

Note: The mean values in the table represent the average of all items in each dimension, and the standard deviations correspond to the means.

**Table 2:** Correlation analysis results.

Variables	(1)	(2)	(3)	(4)	(5)	(6)	(7)	(8)	(9)	(10)
(1) Career education skills	1.000									
(2) Career adaptability	0.596 ***	1.000								
(3) CDMSE	0.633 ***	0.785 ***	1.000							
(4) Gender	−0.086	−0.050	−0.129 *	1.000						
(5) Individual perception	0.848 ***	0.568 ***	0.552 ***	0.038	1.000					
(6) Career planning	0.948 ***	0.528 ***	0.591 ***	0.103	0.636 ***	1.000				
(7) Concern	0.545 ***	0.883 ***	0.667 ***	0.091	0.543 ***	0.468 ***	1.000			
(8) Control	0.531 ***	0.861 ***	0.700 ***	0.047	0.481 ***	0.485 ***	0.711 ***	1.000		
(9) Curiosity	0.537 ***	0.894 ***	0.699 ***	0.008	0.470 ***	0.500 ***	0.690 ***	0.693 ***	1.000	
(10) Confidence	0.481 ***	0.876 ***	0.694 ***	0.026	0.496 ***	0.403 ***	0.683 ***	0.632 ***	0.765 ***	1.000

Note: CDMSE represents career decision-making self-efficacy; *** *p* < 0.01, * *p* < 0.1.

**Table 3:** Mediation model results.

Pathways	Estimate	95%CI		S.E.	Estimate/S.E.	*p*-Value
		Lower	Upper			
Direct effects						
Career education skills → Career adaptability	0.259	0.025	0.559	0.133	1.944	0.052
Indirect effects						
Career education skills → Career decision-making self-efficacy → Career adaptability	0.461	0.286	0.647	0.095	4.867	0.000

## Data Availability

Data will be made available upon request.

## References

[1] Biemann T., Zacher H., Feldman D.C. (2012). Career patterns: A twenty-year panel study. J. Vocat. Behav..

[2] Coetzee M., Harry N. (2014). Emotional intelligence as a predictor of employees’ career adaptability. J. Vocat. Behav..

[3] Puffer K.A. (2011). Emotional Intelligence as a Salient Predictor for Collegians’ Career Decision Making. J. Career Assess..

[4] Chen H., Liu F., Wen Y. (2023). The Influence of College Students’ Core Self-evaluation on Job Search Outcomes: Chain Mediating Effect of Career Exploration and Career Adaptability. Curr. Psychol..

[5] Akkermans J., Richardson J., Kraimer M.L. (2020). The Covid-19 crisis as a career shock: Implications for careers and vocational behavior. J. Vocat. Behav..

[6] Mahmud M.S., Talukder M.U., Rahman S.M. (2021). Does ‘Fear of COVID-19’ trigger future career anxiety? An empirical investigation considering depression from COVID-19 as a mediator. Int. J. Soc. Psychiatry.

[7] Liu X., Guo Y.-X., Xu Y. (2023). Risk factors and digital interventions for anxiety disorders in college students: Stakeholder perspectives. World J. Clin. Cases.

[8] Liu X., Cao X., Gao W. (2022). Does Low Self-Esteem Predict Anxiety Among Chinese College Students?. Psychol. Res. Behav. Manag..

[9] Cao X., Liu X. (2023). Time Use and Cognitive Achievement among Adolescents in China: Depression Symptoms as Mediators. J. Intell..

[10] Cao X. (2023). Sleep Time and Depression Symptoms as Predictors of Cognitive Development Among Adolescents: A Cross-Lagged Study From China. Psychol. Rep..

[11] Tomlinson J., Baird M., Berg P., Cooper R. (2018). Flexible careers across the life course: Advancing theory, research and practice. Hum. Relat..

[12] McDonald P.K. (2018). How ‘flexible’ are careers in the anticipated life course of young people?. Hum. Relat..

[13] Liu X., Ji X., Zhang Y., Gao W. (2023). Professional Identity and Career Adaptability among Chinese Engineering Students: The Mediating Role of Learning Engagement. Behav. Sci..

[14] van Hooft E.A.J., Kammeyer-Mueller J.D., Wanberg C.R., Kanfer R., Basbug G. (2021). Job search and employment success: A quantitative review and future research agenda. J. Appl. Psychol..

[15] Guan Y., Deng H., Sun J., Wang Y., Cai Z., Ye L., Fu R., Wang Y., Zhang S., Li Y. (2013). Career adaptability, job search self-efficacy and outcomes: A three-wave investigation among Chinese university graduates. J. Vocat. Behav..

[16] Phillips S.D. (2015). Lifespan career development. APA Handbook of Career Intervention, Volume 1: Foundations.

[17] Savickas M.L., Porfeli E.J. (2012). Career Adapt-Abilities Scale: Construction, reliability, and measurement equivalence across 13 countries. J. Vocat. Behav..

[18] Savickas M.L. (2013). Career construction theory and practice. Career Dev. Couns. Putt. Theory Res. Work.

[19] Super D.E., Knasel E.G. (1981). Career Development in Adulthood: Some Theoretical Problems and a Possible Solution. Br. J. Guid. Couns..

[20] Cai Z., Tian Y., Wang Z. (2022). Career adaptability and proactive work behaviour: A relational model. J. Occup. Organ. Psychol..

[21] Zacher H. (2016). Within-Person Relationships between Daily Individual and Job Characteristics and Daily Manifestations of Career Adaptability. J. Vocat. Behav..

[22] Coetzee M., Stoltz E. (2015). Employees’ satisfaction with retention factors: Exploring the role of career adaptability. J. Vocat. Behav..

[23] Savickas M.L. (1997). Career adaptability: An integrative construct for life-span, life-space theory. Career Dev. Q..

[24] Savickas M.L. (2005). The Theory and Practice of Career Construction. Career Development and Counseling: Putting Theory and Research to Work.

[25] Pan J.Z., Guan Y.J., Wu J.R., Han L.H., Zhu F., Fu X.Y., Yu J.M. (2018). The interplay of proactive personality and internship quality in Chinese university graduates’ job search success: The role of career adaptability. J. Vocat. Behav..

[26] Mittal S. (2021). Ability-based emotional intelligence and career adaptability: Role in job-search success of university students. High. Educ. Ski. Work-Based Learn..

[27] Fiori M., Bollmann G., Rossier J. (2015). Exploring the path through which career adaptability increases job satisfaction and lowers job stress: The role of affect. J. Vocat. Behav..

[28] Chan S.H.J., Mai X. (2015). The relation of career adaptability to satisfaction and turnover intentions. J. Vocat. Behav..

[29] Zacher H. (2014). Career adaptability predicts subjective career success above and beyond personality traits and core self-evaluations. J. Vocat. Behav..

[30] Zacher H. (2015). Daily manifestations of career adaptability: Relationships with job and career outcomes. J. Vocat. Behav..

[31] Koen J., Klehe U.C., Van Vianen A.E.M. (2012). Training career adaptability to facilitate a successful school-to-work transition. J. Vocat. Behav..

[32] Liu X., Zhang Y., Cao X., Gao W. (2023). Does anxiety consistently affect the achievement goals of college students? A four-wave longitudinal investigation from China. Curr. Psychol..

[33] Al-Waqfi M.A., Tlaiss H., Ghoudi K. (2023). Career Adaptability as a Predictor of Job Search Intentions and Career Readiness of Young Adults in the United Arab Emirates. J. Career Dev..

[34] Hirschi A., Niles S.G., Akos P. (2011). Engagement in adolescent career preparation: Social support, personality and the development of choice decidedness and congruence. J. Adolesc..

[35] Haenggli M., Hirschi A. (2020). Career adaptability and career success in the context of a broader career resources framework. J. Vocat. Behav..

[36] Lee P.C., Xu S., Yang W. (2021). Is career adaptability a double-edged sword? The impact of work social support and career adaptability on turnover intentions during the COVID-19 pandemic. Int. J. Hosp. Manag..

[37] Chan S.H.J., Mai X., Kuok O.M.K., Kong S.H. (2016). The influence of satisfaction and promotability on the relation between career adaptability and turnover intentions. J. Vocat. Behav..

[38] Sibunruang H., Garcia P.R.J.M., Tolentino L.R. (2016). Ingratiation as an adapting strategy: Its relationship with career adaptability, career sponsorship, and promotability. J. Vocat. Behav..

[39] Kuron L.K.J., Lyons S.T., Schweitzer L., Ng E.S.W. (2015). Millennials’ work values: Differences across the school to work transition. Pers. Rev..

[40] Hu X.Y., He Y.Q., Ma D.Y., Zhao S.H., Xiong H., Wan G.B. (2021). Mediating Model of College Students’ Proactive Personality and Career Adaptability. Career Dev. Q..

[41] Liu X., Guo Y.-X., Zhang W.-J., Gao W.-J. (2022). Influencing factors, prediction and prevention of depression in college students: A literature review. World J. Psychiatry.

[42] Wibowo D., Ambarwati K., Crescenzo P. (2021). The role of grit and parent-child communication in career adaptability. Psikohumaniora J. Penelit. Psikol..

[43] Gysbers N.C. (2013). Career-Ready Students: A Goal of Comprehensive School Counseling Programs. Career Dev. Q..

[44] Sullivan S.E., Al Ariss A. (2021). Making sense of different perspectives on career transitions: A review and agenda for future research. Hum. Resour. Manag. Rev..

[45] Blokker R., Akkermans J., Marciniak J., Jansen P., Khapova S. (2023). Organizing School-to-Work Transition Research from a Sustainable Career Perspective: A Review and Research Agenda. Work Aging Retire..

[46] Lent R.W., Morris T.R., Penn L.T., Ireland G.W. (2019). Social-Cognitive Predictors of Career Exploration and Decision-Making: Longitudinal Test of the Career Self-Management Model. J. Couns. Psychol..

[47] Chen S., Chen H.R., Ling H.R., Gu X.Y. (2021). How Do Students Become Good Workers? Investigating the Impact of Gender and School on the Relationship between Career Decision-Making Self-Efficacy and Career Exploration. Sustainability.

[48] Tolentino L.R., Garcia P.R.J.M., Lu V.N., Restubog S.L.D., Bordia P., Plewa C. (2014). Career adaptation: The relation of adaptability to goal orientation, proactive personality, and career optimism. J. Vocat. Behav..

[49] Cai Z., Guan Y., Li H., Shi W., Guo K., Liu Y., Li Q., Han X., Jiang P., Fang Z. (2015). Self-esteem and proactive personality as predictors of future work self and career adaptability: An examination of mediating and moderating processes. J. Vocat. Behav..

[50] Jiang Z. (2017). Proactive personality and career adaptability: The role of thriving at work. J. Vocat. Behav..

[51] Li Y., Guan Y., Wang F., Zhou X., Guo K., Jiang P., Mo Z., Li Y., Fang Z. (2015). Big-five personality and BIS/BAS traits as predictors of career exploration: The mediation role of career adaptability. J. Vocat. Behav..

[52] Oztemel K., Yildiz-Akyol E. (2021). The Predictive Role of Happiness, Social Support, and Future Time Orientation in Career Adaptability. J. Career Dev..

[53] Du B., Yu X., Luo N., Liu X.H. (2022). The effect of core self-evaluations on career adaptability: The mediating role of protean career attitudes and the moderating role of meritocratic beliefs. Front. Psychol..

[54] Guan P.P., Capezio A., Restubog S.L.D., Read S., Lajom J.A.L., Li M. (2016). The role of traditionality in the relationships among parental support, career decision-making self-efficacy and career adaptability. J. Vocat. Behav..

[55] Song Y., Mu F., Zhang J.H., Fu M.C. (2022). The Relationships Between Career-Related Emotional Support From Parents and Teachers and Career Adaptability. Front. Psychol..

[56] Garcia P.R.J.M., Restubog S.L.D., Ocampo A.C., Wang L., Tang R.L. (2019). Role modeling as a socialization mechanism in the transmission of career adaptability across generations. J. Vocat. Behav..

[57] Cheung R., Jin Q.P. (2016). Impact of a Career Exploration Course on Career Decision Making, Adaptability, and Relational Support in Hong Kong. J. Career Assess..

[58] Savickas M.L. (2012). Life Design: A Paradigm for Career Intervention in the 21st Century. J. Couns. Dev..

[59] Helens-Hart R. (2019). Career Education Discourse: Promoting Student Employability in a University Career Center. Qual. Res. Educ..

[60] Lam M., Santos A. (2018). The Impact of a College Career Intervention Program on Career Decision Self-Efficacy, Career Indecision, and Decision-Making Difficulties. J. Career Assess..

[61] Carvalho L., Moura L., Freitas C. (2023). Career counseling for college students: Assessment of an online and group intervention. J. Vocat. Behav..

[62] Branan J., Li X.R., Wheeler R. (2018). BUILDING a career planning course for STEM PhDs. Nat. Biotechnol..

[63] Dozier V.C., Morgan M., Burbrink I., Peace C. (2022). Standardized career course curriculum: Effects on negative career thoughts. Career Dev. Q..

[64] Gu X.Y., Tang M., Chen S., Montgomery M.L.T. (2020). Effects of a Career Course on Chinese High School Students’ Career Decision-Making Readiness. Career Dev. Q..

[65] Bandura A. (1986). Social Foundations of Thought and Action: A Social Cognitive Theory.

[66] Bandura A. (1977). Self-efficacy: Toward a unifying theory of behavioral change. Psychol. Rev..

[67] Taylor K.M., Betz N.E. (1983). Applications of self-efficacy theory to the understanding and treatment of career indecision. J. Vocat. Behav..

[68] Betz N.E., Klein K.L., Taylor K.M. (1996). Evaluation of a short form of the Career Decision-Making Self-Efficacy Scale. J. Career Assess..

[69] Liu X., Ji X., Zhang Y. (2023). Trajectories of college students’ general self-efficacy, the related predictors, and depression: A piecewise growth mixture modeling approach. Heliyon.

[70] Santos A., Wang W., Lewis J. (2018). Emotional intelligence and career decision-making difficulties: The mediating role of career decision self-efficacy. J. Vocat. Behav..

[71] Boo S., Wang C., Kim M. (2021). Career adaptability, future time perspective, and career anxiety among undergraduate students: A cross-national comparison. J. Hosp. Leis. Sport Tour. Educ..

[72] Garcia P.R.J.M., Restubog S.L.D., Bordia P., Bordia S., Roxas R.E.O. (2015). Career optimism: The roles of contextual support and career decision-making self-efficacy. J. Vocat. Behav..

[73] Rudolph C.W., Lavigne K.N., Zacher H. (2017). Career adaptability: A meta-analysis of relationships with measures of adaptivity, adapting responses, and adaptation results. J. Vocat. Behav..

[74] Stead G.B., LaVeck L.M., Rua S.M.H. (2022). Career Adaptability and Career Decision Self-Efficacy: Meta-Analysis. J. Career Dev..

[75] Jiang R.Y., Fan R.M., Zhang Y., Li Y.X. (2022). Understanding the serial mediating effects of career adaptability and career decision-making self-efficacy between parental autonomy support and academic engagement in Chinese secondary vocational students. Front. Psychol..

[76] Hamzah S.R., Le K.K., Musa S.N.S. (2021). The mediating role of career decision self-efficacy on the relationship of career emotional intelligence and self-esteem with career adaptability among university students. Int. J. Adolesc. Youth.

[77] Hou C.N., Wu Y.Z., Liu Z.J. (2019). Career decision-making self-efficacy mediates the effect of social support on career adaptability: A longitudinal study. Soc. Behav. Personal..

[78] Aslan Y., Koçak O. (2023). How do family influence on career choices and perceived social support affect students’ life satisfaction in Turkey? The role of vocational outcome expectations as mediator. Int. J. Educ. Vocat. Guid..

[79] Reese R.J., Miller C.D. (2006). Effects of a university career development course on career decision-making self-efficacy. J. Career Assess..

[80] Kim J.H., Shin H.S. (2020). Effects of self-reflection-focused career course on career search efficacy, career maturity, and career adaptability in nursing students: A mixed methods study. J. Prof. Nurs..

[81] Yu H., Dong Z., Guan X., Yan C., Su X., Cheng L. (2022). A Multiple Mediational Meta-Analysis of the Influence of Proactive Personality on Subjective Career Success at the Career Exploration Stage. J. Career Assess..

[82] Wright S., Perrone K. (2010). An Examination of the Role of Attachment and Efficacy in Life Satisfaction. Couns. Psychol..

[83] Li H., Ngo H.-y., Cheung F. (2019). Linking protean career orientation and career decidedness: The mediating role of career decision self-efficacy. J. Vocat. Behav..

[84] Hu L.t., Bentler P.M. (1999). Cutoff criteria for fit indexes in covariance structure analysis: Conventional criteria versus new alternatives. Struct. Equ. Model. A Multidiscip. J..

[85] Wong L.P.W., Yuen M., Chen G. (2019). Technology-Infused Career and Life Planning Education. Asia Pac. Career Dev. J..

[86] Zhai C.X., Chai X.Y., Shrestha S., Zhong N. (2023). Grit and Career Construction among Chinese High School Students: The Serial Mediating Effect of Hope and Career Adaptability. Sustainability.

[87] Ling H., Teng S., Liu X., Wu J., Gu X. (2022). Future Work Self Salience and Future Time Perspective as Serial Mediators Between Proactive Personality and Career Adaptability. Front. Psychol..

[88] Green Z., Noor U., Hashemi M. (2020). Furthering Proactivity and Career Adaptability Among University Students: Test of Intervention. J. Career Assess..

[89] Welde A.M.J., Bernes K.B., Gunn T.M., Ross S.A. (2015). Integrated career education in senior high: Intern teacher and student recommendations. Aust. J. Career Dev..

[90] Parmentier M., Pirsoul T., Nils F. (2019). Examining the impact of emotional intelligence on career adaptability: A two-wave cross-lagged study. Personal. Individ. Differ..

[91] Romero-Rodriguez S., Figuera-Gazo P., Freixa-Niella M., Llanes-Ordonez J. (2019). Career adaptability among University students: A study through autobiographical interviews. Rie-Rev. De Investig. Educ..

[92] Liu X.Q., Guo Y.X., Wang X. (2023). Delivering substance use prevention interventions for adolescents in educational settings: A scoping review. World J Psychiatry.

